# Comparison of hydroxyethyl starch regulatory summaries from the Food and Drug Administration and the European Medicines Agency

**DOI:** 10.1186/s40545-016-0090-6

**Published:** 2017-03-21

**Authors:** Christian J. Wiedermann, Klaus Eisendle

**Affiliations:** 1 Department of Internal Medicine, Central Hospital of Bolzano, Lorenz-Böhler-Street 5, 39100 Bolzano, BZ Italy; 2 Department of Research, College of Health Professions Claudiana, Bolzano, Italy

**Keywords:** Volume resuscitation, Drug safety, Drug regulatory affairs, Hydroxyethyl starch, Colloids

## Abstract

This article aims to highlight the positions of the Food and Drug Administration and the European Medicines Agency regarding use and marketing of hydroxyethyl starch (HES) products, and how these have changed over recent years. In 2013, warnings from both agencies advised against use of HES in critically ill patients, including patients with sepsis, when several large randomized controlled trials on volume resuscitation in critical illness failed to observe clinically beneficial effects of HES. In areas such as patient monitoring and requirements for further clinical trials, the FDA and EMA are very much in agreement in their recommendations. However, EMA guidance is generally more restrictive on HES usage compared to that from the FDA. Differences in data presented to regulatory authorities, bias in study results and inherent weakness of meta-analyses used for drug surveillance purposes, plus different risk-management approaches used by the two regulatory authorities, likely contribute to different outcomes in their regulations concerning use of HES.

## Background

The Food and Drug Administration (FDA) and the European Medicines Agency (EMA) are two of the most influential drug regulatory authorities in the world, and are responsible for reviewing and regulating biomedical products and supervising clinical trials, marketing approvals, and risk-management processes. The FDA supervises these processes in the United States of America, whereas in the European Union this is overseen in a coalition of federal organisations that includes the EMA, the European Commission, and the national authorities of the member states. The EMA and FDA have different evaluation processes; therefore, despite the submission of identical clinical data supporting the same drug, the two bodies can come to different evaluations and conclusions [1].

### Purpose

This document aims to highlight the summary positions of the FDA and EMA regarding use and marketing of hydroxyethyl starch (HES) products, and how these have changed over recent years. HES is an artificial colloid used worldwide for volume resuscitation [2]. Regulatory decisions regarding HES are controversial [3], partly due to use of meta-analyses for safety evaluations, which is known to be problematic [4].

## Method

Source information for this summary was retrieved from the FDA and EMA websites [5, 6] using the following general search terms: ‘HES’, ‘hydroxyethyl starch’, ‘tetrastarch’, and ‘hetastarch’, plus specific product names: Hespan, Hextend, Voluven and Tetraspan. An overview of events in HES usage between 2010 to present day is shown in Table 1. Changes in regards to labelling and recommendations issued by the FDA and EMA, including dosage, indications, contraindications, adverse effects, warnings, precautions, and patient monitoring are summarized in Table 2.

**Table 1:** Timeline of HES regulatory key events between 2010 to current practice

*Safety concern*	*2010 – Publication of Cochrane review of HES effects on kidney function. Increased risk in septic shock patients with 10% HES 200/0.5 or 6% HES 200/0.6* [28] *(Not based on US studies, i.e. HES formulations studied are different from those FDA approved.* *2012 – Publication of CRYSTMAS (May 2012), 6S (Jul 2012), & CHEST (Nov 2012) trials (plus others)* [12–14]	
Timeline	FDA	EMA
Before 2010	1972 – Approval of HESPAN (6% HES 450/0.7) 1991 – Approval of Hetastarch (6% HES 450/0.7) 1999 – Approval of Hextend (6% HES 450/0.7) 2007 – Approval of Voluven (6% HES 130/0.4)	HES-containing medicinal products (>60 available throughout Europe) in use for decades [29]
2012 May	Change to Voluven labelling to include increased frequency and duration of renal replacement therapy among Voluven patients and info on paediatric dosage *(in response to CRYSTMAS study)* [30]	
2012 Sept	Expert workshop set up by FDA to discuss HES products in light of recent data [27]	
2012 Nov		Article 31 referral received following concerns over safety of HES products – review conducted by PRAC [31]
2013 Jun		PRAC recommends suspending marketing authorisations (MA) for all HES products and their use in all patient populations [32]
2013 Jun		MA holders appeal against decision [32]
2013 Oct		PRAC revises recommendation upon completion of review. HES solutions may continue to be used in restricted patient population and additional studies should be conducted [16]
2013 Oct		CMDh endorses PRAC recommendations, decision sent to European Commission [16]
2013 Nov	Addition of black box warning to product information regarding increased mortality and kidney injury in critically ill patients [28]	
2013 Nov	Additional warning about excessive bleeding needed in the Warnings and Precautions Section of the package insert - considered a class effect [27]	
2013 Nov	Voluven label change: “Severe liver disease” added to contraindication due to data from CHEST trial [30]	
2013 Dec		EU-wide decision to allow HES product use in restricted patient population [16]
2014 Mar		Revised product information formally issued across entire EU [16]
2014 Sept	Additional precaution added to paediatric use section (Voluven) [33]	
2014 Oct		PASS protocol rejected by PRAC due to issues with the study design [34]
2015 Mar		PASS protocol approved by PRAC [35]
2015 Jul		Another PASS protocol rejected by PRAC due to inadequate sample size [36] Benefits of HES products in approved indications remain favourable. No changes to labelling or MA [36]
2015 Oct		PRAC issued advice and recommendations (requested by EU member states) regarding submitted PASS protocols [37]
2015 Nov		PASS protocol approved by PRAC [38]
Current	Two post-marketing commitments fulfilled (Voluven) [13, 39, 40] Routine surveillance using FAERS.	HES products listed under ‘Medicines under additional monitoring’ by EMA since 2013 as further PASS required and all data reviewed periodically (PSUR) Post-authorisation commitment to perform additional studies in patients with trauma and in elective surgery [16] MAH (Serum Werke Bernburg) HES products licence suspension for 1 year from Sept 2015 to Sept 2016 [41] Further, PASS protocol submitted to be discussed at Sept 2016 meeting [42]
Future		Use of HES in perioperative settings and the potential dose dependency of HES administration in relation to acute kidney injury to be assessed [36]

*Abbreviations*: *CMDh* Coordination Group for Mutual recognition and decentralised Procedures – human, *FAERS* FDA Adverse Event Reporting System, *MAH* Marketing Authorisation Holder, *PASS* Post-Authorisation Safety Study, *PRAC* Pharmacovigilance Risk Assessment Committee, *PSUR* Periodic Safety Update Report

**Table 2:** Changes to guidance on HES products from the FDA and EMA between 2012 and 2016

Variable	FDA	EMA
Dosage	6% Hetastarch Adults Administer 500 to 1000 mL. Doses of more than 1500 mL/day for the typical 70 kg patient (approx. 20 mL/kg) are usually not required although doses of isotonic solutions containing 6% hetastarch up to 1500 mL have been used during major surgery. Volumes in excess of 1500 mL/day have been used where severe blood loss has occurred. Newborns and children Adequate, well controlled clinical trials to establish the safety and effectiveness of HEXTEND in paediatric patients have not been conducted.	6% Hetastarch Identical to FDA
	6% Tetrastarches Adults Administer up to 30 mL/kg/day. This dose is equivalent to 2100 mL for a 70 kg patient. Give initial 10–20 mL by slow infusion and monitor for adverse effects. Newborns and children Limited data on the use of 130/0.4 HES in children is available - if used individualised dose taking into account disease and haemodynamic status should be employed.	6% Tetrastarches Identical to FDA
	*No change to adult dosage guidelines in 2012* *Extra information to Voluven label in 2012* <2 years: Mean dose of 16 mL/kg IV 2–12 years: Mean dose of 36 mL/kg IV	*No change to adult dosage guidelines in 2012* *Extra information to Voluven label in 2012* <2 years: Mean dose of 16 mL/kg IV 2–12 years: Mean dose of 36 mL/kg IV
Indications	Hetastarches indicated for treatment of hypovolaemia when plasma volume expansion is desired. It is not a substitute for blood or plasma.	Treatment of imminent or manifest hypovolaemia and shock.
	*No changes to indication text in 2012*	*Change to indication text in 2013* In case of hypovolaemia a crystalloid solution should first be given. HES is indicated for the treatment of hypovolaemia if patient does not respond to crystalloid solution.
Contra-indications	Renal failure (with oliguria or anuria) Hypersensitivity Congestive cardiac failure Treatment of lactic acidosis Patients on dialysis Clinical conditions with volume overload	Renal failure (with oliguria or anuria) Hypersensitivity Congestive cardiac failure Hyperhydration states including pulmonary edema Intracranial bleeding Severely impaired hepatic function Hyperkalaemia Severe hypernatremia or hyperchloremia Clinical conditions with volume overload
	*Additional contraindications in 2013* Critically ill adult patients, including patients with sepsis, and those admitted to the ICU Pre-existing renal function Severe liver disease Patients with pre-existing coagulation and bleeding disorders	*Additional contraindications in 2013* Critically ill patients, including patients with sepsis Burn injuries Renal impairment or renal replacement therapy patients Severe coagulopathy Organ transplant patients
Adverse Effects	*Additions to adverse effects in 2013* Increased mortality in severe sepsis and other conditions requiring ICU admission Increased need for RRT in severe sepsis and other conditions requiring ICU admission	*Additions to adverse effects in 2013* Renal injury Hepatic injury
Warnings and Precautions	*Black box warning added to label in 2013* Avoid use in patients with pre-existing renal dysfunction Discontinue use of HES at the first sign of renal injury Avoid fluid overload; adjust dosage with cardiac or renal dysfunction In cases of severe dehydration, a crystalloid solution should be given first	*Restriction on patient population added to label in 2013* Only use HES during the first 24 hours of fluid resuscitation treatment Trauma and surgery: carefully weigh the expected benefit of treatment against the uncertainty of the long term safety of treatment. Consider other available treatment options Stop HES treatment at the first sign of impaired blood clotting or renal injury Not recommended for patients undergoing open heart surgery
Patient Monitoring	Monitor fluid balance, serum electrolytes, renal and hepatic function, acid–base balance, and coagulation parameters during prolonged parenteral therapy or when warranted	Because of the risk of allergic (anaphylactoid) reactions, the patient should be monitored closely and the infusion instituted at a low rate Monitor fluid balance, serum electrolytes, renal and hepatic function. Particular caution in patients with renal impairment and elderly patients Elevated serum amylase levels may be observed and can interfere with the diagnosis of pancreatitis
	*Additions to guidance in 2013* Monitor renal function in hospitalized patients for at least 90 days as use of renal replacement therapy has been reported up to 90 days after administration of HES products Monitor the coagulation status of patients undergoing open heart surgery in association with cardiopulmonary bypass as excess bleeding has been reported with HES solutions in this population. Discontinue use of HES at the first sign of coagulopathy Monitor liver function in patients receiving HES products	*Additions to guidance in 2013* Monitor kidney function in patients receiving HES for at least 90 days and stop HES treatment at the first sign of renal injury Blood coagulation parameters should be monitored carefully in case of repeated administration HES solutions should be used at the lowest effective dose for the shortest period of time Treatment should be guided by continuous haemodynamic monitoring so that the infusion is stopped as soon as appropriate haemodynamic goals have been achieved

### FDA and EMA policy changes

A key event in the regulation of HES occurred in 2013, when both agencies advised against its use in critically ill patients, including patients with sepsis. Several large randomized controlled trials (RCTs) on volume resuscitation in critical illness with low risk of bias failed to observe clinically beneficial effects of HES (for a review, see [7]) and confirmed previous doubts regarding the safety profile of tetrastarches [8]. This included an association between colloid exposure and morbidity, including acute renal failure in various clinical settings [9]. Moreover, randomized trials and meta-analyses have demonstrated that HES increases the need of renal replacement therapy, including in surgical patients [10], with tissue storage of HES cited as a likely mechanism of toxicity [11].

Publication of these and other pivotal studies led the FDA and EMA to instigate a review of HES product usage. In September 2012 the FDA convened a workshop with experts from academia, industry, and other relevant stakeholder to discuss the risks and benefits of HES products. Similarly, in November 2012, a review was opened by the EMA at the request of the German medicines agency, following concerns relating to the safety of HES products. During these reviews both agencies reviewed the data from RCTs, meta-analyses and observational studies, with a particular emphasis on 6S, CRYSTMAS (FDA only), CHEST and VISEP studies (EMA only) [12–15].

The outcome of these reviews led to several key regulatory decisions by the FDA and EMA, as outlined in Table 1. These included the FDA issuing new safety information as a black box warning (Nov 2013), and the suspension and subsequent limitations of marketing and use of HES products in restricted patient populations by the EMA (June 2013; Oct 2013) [16]. In addition, the FDA and EMA also updated their guidance on dosage, indication, contraindication, adverse effects, warning, precautions and patient monitoring in relation to the use of HES products (Table 2). Generally, the EMA restricts use of HES to a greater extent than the FDA. In areas such as patient monitoring and the requirement for further HES clinical trials, the FDA and EMA are very much in agreement; however, differences in contraindications are evident, which suggests that risk-management approaches used by the two regulatory authorities differ. The EMA oversees more than 60 HES products currently on the market within the EU and is required to work with each member states’ regulatory authority; this added complexity can inherently result in more restrictive measures when compared to the FDA. For example, the FDA and EMA definitions of conditions can vary, such as for hypovolemia where the FDA definition is broad, whereas the EMA specify different types.

### Data quality

Unpublished and misreported studies make it difficult for regulatory bodies to determine the true value of a treatment [17]. The CHEST trial [14], a large-scale, randomised controlled trial to evaluate the safety and efficacy of 6% HES in ICU patients, erroneously misreported safety data on pruritus induced by HES vs. saline, which was considered serious enough by the editors of the *NEJM* that a corrigendum was published [18]. This may be significant because the true value of HES remains controversial [7, 19] and pruritus is among its known side effects [11]. Fresenius Kabi, a major manufacturer of tetrastarch and other HES products, as well as a funder of the CHEST trial, questioned the trial’s reporting in the *NEJM.* The *British Medical Journal* finally published on the dispute between Fresenius Kabi and the CHEST investigators [20, 21] as part of the open data campaign [22].

Regulatory authorities’ risk-management approaches include thorough analyses of results from RCTs. Regarding regulatory summaries for HES, selective outcome reporting has previously been identified in publications of the CRYSTMAS and FIRST trials [23]. For example, in the CRYSTMAS trial, Fresenius Kabi was involved in the study design, analysis and preparation of the report [23]. Also, a meta-analysis of RCTs on use of HES for volume resuscitation in cardiac surgery concluded that, as compared to other volume resuscitation solutions, HES had no adverse effects on blood loss, transfusion requirements, and length of hospital stay [24]. The publication’s addendum states that Fresenius Kabi presented the meta-analysis to the EMAs PRAC as part of obligations arising under Article 31 and to fulfil Article 107i of the process regarding the perioperative use of HES. In addition, the authors state that statistical analysis had been conducted by MARCO and had been provided by Fresenius Kabi [24]. The conclusion of lower blood loss with tetrastarch than with pentastarch from this study is attributable to publication bias, since an unpublished trial with higher blood loss and more frequent reoperation for bleeding after tetrastarch was omitted from this meta-analysis [24]. Conversely, it was included in a meta-analysis from 2012 [24] on the same indication, which found that HES products increase postoperative blood loss, need for blood product transfusions, and need for re-operations [25, 26]. Findings from the 2012 meta-analysis contributed to the FDA’s decision to issue a security warning because of excessive bleeding as a class effect of all HES solutions (boxed warning for the use of HES [27]). Incidentally, the tetrastarch manufacturer who commissioned the 2014 meta-analysis [21] had previously submitted the unpublished trial (study No. HS-13-24-EN in the 2012 meta-analysis [25]) to the FDA in a New Drug Application. This observation supports the hypothesis that diverging summaries of the FDA versus the EMA may be based on different type and quality of data that drug manufacturers present to regulatory authorities.

## Conclusions

A significant change to guidelines regarding use of HES for volume replacement was introduced in 2013 by both the FDA and EMA, warning of increased risk of death and renal injury and advising against use of HES in critically ill patients, including patients with sepsis. Both agencies have adopted a similar stance regarding HES usage, but the EMA restricts use to a greater extent. For example, the EMA warning also includes burn injuries as a contraindication, and includes additional advice stating crystalloids should be the first-line treatment; HES should be used only where crystalloids alone are insufficient, and only then for short periods of time.

In areas such as patient monitoring and the requirement for further HES clinical trials, guidelines from the FDA and EMA are generally in agreement. Slight variations exist regarding dosage recommendations, but the majority of HES products used in the USA are hetastarches, whereas in Europe tetrastarches appear to be predominant.

## Abbreviations

CMDh: Coordination Group for Mutual Recognition and Decentralised Procedures – human
EMA: European Medicines Agency
FAERS: FDA Adverse Event Reporting System
FDA: Food and Drug Administration
HES: Hydroxyethyl Starch
MAH: Marketing Authorisation Holder
PASS: Post-Authorisation Safety Study
PRAC: Pharmacovigilance Risk Assessment Committee
PSUR: Periodic Safety Update Report
RCT: Randomized Controlled Trial
